# The ω-3 Polyunsaturated Fatty Acid, Eicosapentaenoic Acid, Attenuates Abdominal Aortic Aneurysm Development via Suppression of Tissue Remodeling

**DOI:** 10.1371/journal.pone.0096286

**Published:** 2014-05-05

**Authors:** Jack H. Wang, Kosei Eguchi, Sahohime Matsumoto, Katsuhito Fujiu, Issei Komuro, Ryozo Nagai, Ichiro Manabe

**Affiliations:** 1 Department of Cardiovascular Medicine, Graduate School of Medicine, The University of Tokyo, Tokyo, Japan; 2 Jichi Medical University, Tochigi, Japan; Brigham and Women's Hospital, Harvard Medical School, United States of America

## Abstract

Abdominal aortic aneurysm (AAA) is a prevalent vascular disease that can progressively enlarge and rupture with a high rate of mortality. Inflammation and active remodeling of the aortic wall have been suggested to be critical in its pathogenesis. Meanwhile, ω-3 polyunsaturated fatty acids such as eicosapentaenoic acid (EPA) are known to reduce cardiovascular events, but its role in AAA management remains unclear. Here, we show that EPA can attenuate murine CaCl_2_-induced AAA formation. Aortas from BALB/c mice fed an EPA-diet appeared less inflamed, were significantly smaller in diameter compared to those from control-diet-fed mice, and had relative preservation of aortic elastic lamina. Interestingly, CT imaging also revealed markedly reduced calcification of the aortas after EPA treatment. Mechanistically, MMP2, MMP9, and TNFSF11 levels in the aortas were reduced after EPA treatment. Consistent with this finding, RAW264.7 macrophages treated with EPA showed attenuated *Mmp9* levels after TNF-α simulation. These results demonstrate a novel role of EPA in attenuating AAA formation via the suppression of critical remodeling pathways in the pathogenesis of AAAs, and raise the possibility of using EPA for AAA prevention in the clinical setting.

## Introduction

Abdominal aortic aneurysm (AAA) is a disease that can be defined as the gradual and irreversible dilatation of the abdominal aorta [1]. It is common in men older than 65 years of age and has a reported prevalence of 4–9% in men and 1–2% in women [2]–[5]. Surgical or endovascular repairs continue to be the only definitive treatment options for AAA, whereas pharmacological therapy for the prevention or slowing of AAA formation remains limited. Without treatment, natural disease progression of the AAA results in rupture. Given that the mortality rate for patients with AAA rupture remains very high (65–85%) [5], new forms of pharmacological treatment are needed to improve patient outcomes for this common but silent and deadly disease.

Recent studies have revealed that inflammatory processes play a key role in the development of AAAs, which involves the infiltration of various immune cells (particularly macrophages and T cells) [6]–[8] as well as activation of inflammatory pathways [4], [9], [10]. Importantly, matrix metalloproteinase (MMP) 9 derived from macrophages and MMP2 derived from vascular smooth muscle cells (SMCs) [4]–[6] have been shown to be critical factors required for the elastin destruction and proteolytic degradation that are hallmark features of AAAs, thereby leading to gradual aortic dilatation. Interestingly, such vascular wall degradation in human AAAs is often also accompanied by calcification of the aneurysmal wall, suggesting a possible link between aneurysm formation and calcification [1].

The ω-3 polyunsaturated fatty acids (PUFAs) are a class of essential fatty acids required for normal biological activity and function in living organisms. These fatty acids can typically be either plant-derived (α-linolenic acid) or marine fish-derived [eicosapentaenoic acid (EPA) and docosahexaenoic acid (DHA)] [11]. From numerous clinical, epidemiological, and animal studies, ω-3 PUFAs have been demonstrated to possess anti-inflammatory [12]–[14], anti-fibrotic [15], [16], and cardioprotective properties [11], [17]–[19], and they are already being used widely as pharmacological agents and nutritional supplements in humans. They have been suggested to have various mechanisms of action, including the ability to reduce the production of inflammatory eicosanoids by competing with arachidonic acid (AA) [11], exertion of anti-inflammatory effects via ligand-receptor interactions with the G protein-coupled receptor 120 (GPR120) [13], and activation of the resolution of inflammation by ω-3 PUFA metabolites such as resolvin E1 and protectin D1 [12], [20]. However, the precise molecular mechanisms as to how ω-3 PUFAs exhibit beneficial effects in each pathological process still remain to be elucidated.

The role of ω-3 PUFAs in the management of AAAs has not been established. Given the anti-inflammatory properties of ω-3 PUFAs, we hypothesized that ω-3 PUFA might suppress the formation of AAAs by attenuating tissue remodeling processes. Using CaCl_2_ to induce the development of AAA in mice is a well-established method that recapitulates some of the hallmark features of AAAs, such as inflammation, immune cell infiltration, calcification, and upregulation of tissue remodeling factors [21]. In this study, we show that EPA can attenuate the formation of AAAs in the CaCl_2_-induced AAA model by suppressing tissue remodeling processes.

## Results

### EPA treatment attenuates CaCl_2_-induced AAA formation and elastic lamina destruction

**Figure 1:**
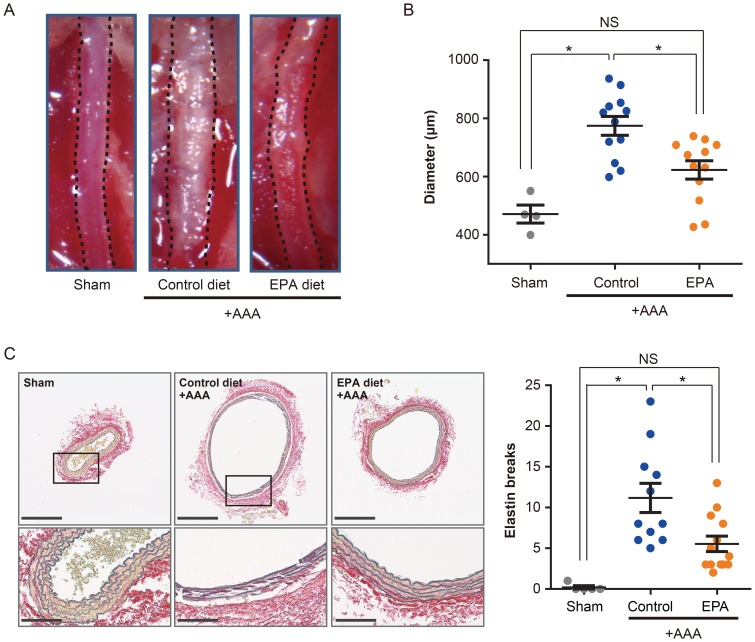
EPA reduces aortic aneurysm formation. Gross morphological and histological analyses of aortas were performed at 6 weeks after perivascular application of CaCl_2_ to the infra-renal aorta. **A**. Representative images of *in situ* infra-renal aortas (demarcated by the broken lines) from mice in the sham-operated, control diet or EPA diet groups. **B**. Quantitative analysis of the maximal external aortic diameters of aortas. *n* = 4 for sham, *n* = 12 for control diet and EPA diet groups. **C**. Histological analysis by EVG staining, showing preserved aortic wall structure of the aorta from EPA diet group compared to the aorta from control diet group. Elastin breaks were also quantified. Scale bars, 200 µm (upper panels) and 50 µm (lower panels). *n* = 5 for sham, *n* = 11 for control diet, and *n* = 12 for EPA diet groups. Representative images of at least three independent experiments are shown in **A** and **C**. *P<0.05.

Abdominal aortic aneurysm formation was induced by CaCl_2_ in BALB/c mice fed either a control or EPA-supplemented diet. Marked dilatation and calcification of the aorta in the control diet group was clearly visible 6 weeks after CaCl_2_ was applied to the infrarenal abdominal aorta; in contrast, the aortas of the mice on the EPA diet were dilated significantly less than those of control mice (Figure 1A, B). The aortic diameters in the control diet group were shown to have significantly increased by approximately 64% compared to sham-treated mice, which meets the definition for aneurysm formation (≥50% increase in aortic diameter [22]), whereas the aortic diameters in the EPA diet group were not significantly different from those of the sham group. Moreover, the aortic diameters of EPA diet group were significantly smaller than those of the control diet group, indicating that EPA treatment attenuated the formation of CaCl_2_-induced AAA (Figure 1B).

Histological examination of the CaCl_2_-treated infra-renal aortas demonstrated that the extensive matrix and elastic lamina destruction seen in control AAAs was greatly suppressed in aortas from the EPA diet group (Figure 1C). Higher magnification views showed that the elastic lamina strand breaks clearly seen in AAAs of the control diet group were relatively absent in the EPA diet group. Consistent with these observations of vascular wall remodeling, medial fibrosis of the vascular wall was also markedly suppressed in the aortas of the EPA diet group compared with those of the control diet group (Figure S1 in File S1). Taken together, the results support the notion that EPA attenuated aortic dilatation via suppression of vascular wall remodeling.

### Aortic calcification was suppressed by EPA

**Figure 2:**
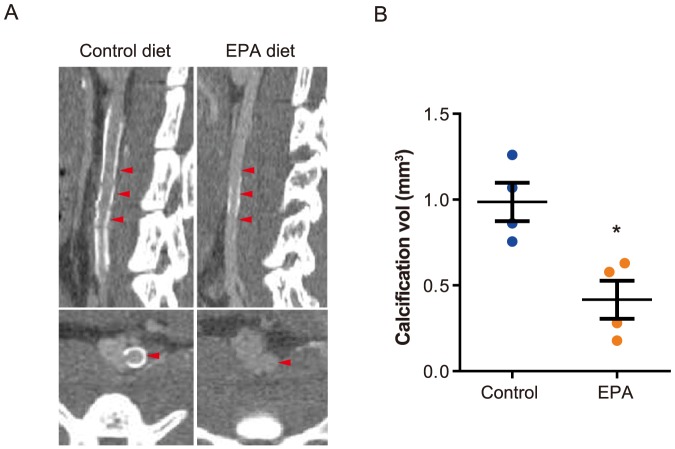
EPA suppressed aortic calcification after AAA-induction. Aortic calcification was assessed by micro-CT imaging of *in situ* aortas 6 weeks after perivascular CaCl_2_ application. Both sagittal and transverse slices (**A**) show reduced overall calcification in the infra-renal aortas from EPA diet group compared to the control diet group, and this was consistent with the results of quantitative analysis of the total calcification volume in each aorta (**B**). *n* = 4 for control diet and EPA diet groups. Red arrowheads indicate the posterior wall of the infra-renal aorta. Representative images of two independent experiments are shown in **A**. *P<0.05 compared to control diet group.

The aortic walls of CaCl_2_-induced AAAs in mice from the control diet group had clear, macroscopically visible calcification, whereas an EPA diet attenuated this macroscopic calcification (Figure 1A). Consistent with this observation, micro-computed tomography (CT) revealed that calcification along the area of the aorta to which CaCl_2_ had been applied was significantly reduced in the EPA diet group compared to the control diet group (Figure 2A, B).

### EPA attenuated the CaCl_2_-induced upregulation of MMPs and the calcification factor RANKL in AAAs

**Figure 3:**
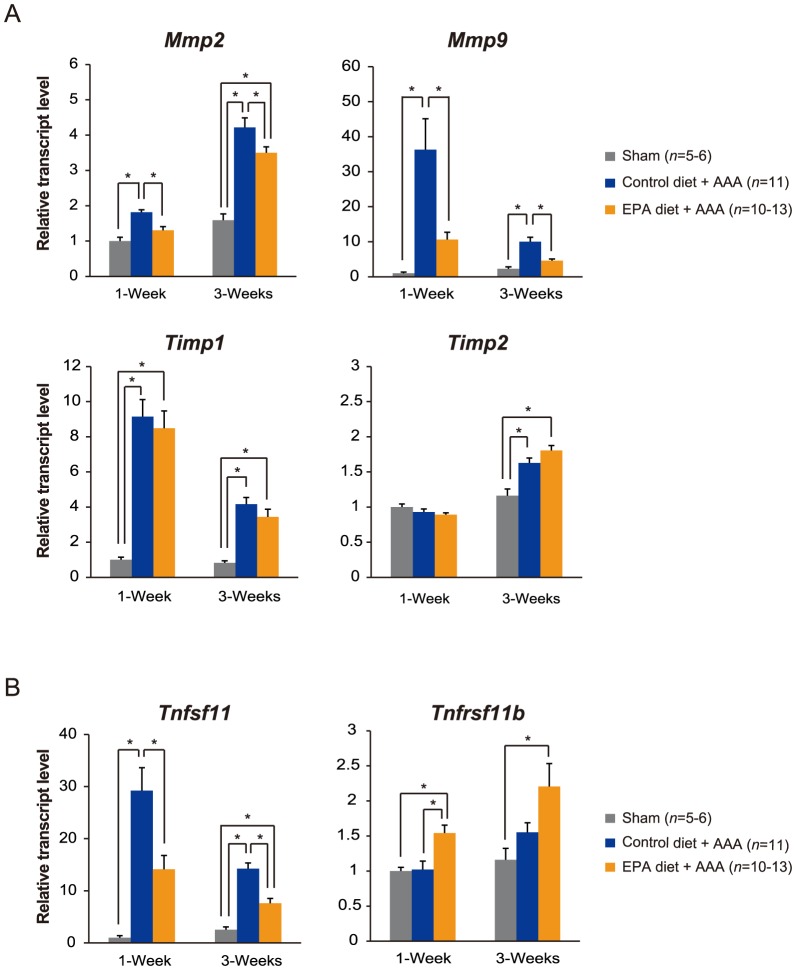
EPA attenuates *Mmp9* and *Tnfsf11* upregulation in CaCl_2_-induced AAA. A . mRNA levels of the matrix metalloproteases *Mmp2* and *Mmp9*, as well as their tissue inhibitors *Timp1* and *Timp2*, in aortas at 1 and 3 weeks after perivascular CaCl_2_ application were analyzed using real-time RT-PCR. **B**. The mRNA levels of the factors known to be involved in the development of vascular calcification, *Tnfsf11* and *Tnfrsf11b*, were also similarly analyzed using real-time RT-PCR. All expression levels were first normalized to *18s* rRNA levels and then presented as fold change over the sham group. *P<0.05.

We subsequently began elucidating the molecular mechanism as to how EPA suppressed AAA formation, by first focusing on the expression of a set of genes related to tissue remodeling. Among the genes analyzed by real-time PCR, the expression levels of the matrix metalloproteinase (MMP) genes *Mmp2* and *Mmp9* were significantly increased in the aortas of control diet-fed mice at 1 and 3 weeks after CaCl_2_ application, consistent with previous reports [6], [9]. In contrast, EPA diet-fed mice had significantly lower levels of *Mmp2* and *Mmp9* expression (Figure 3A). While tissue inhibitor of metalloproteinases (TIMP) *Timp1* and *Timp2* were also upregulated by the CaCl_2_ treatment, EPA did not affect their expression (Figure 3A).

Given that vascular calcification was also reduced by the EPA diet, we next examined the expression levels of factors known to be implicated in this process. A marked upregulation of *Tnfsf11* encoding Receptor Activator of Nuclear Factor κB Ligand (RANKL), a member of the tumor necrosis factor superfamily that is known to be a major factor that increases vascular calcification and maintains bone homeostasis [23], [24], was observed in AAAs of the control diet group after CaCl_2_-induction. Interestingly, EPA diet significantly attenuated *Tnfsf11* upregulation (Figure 3B). In addition, EPA diet increased the expression levels of *Tnfrsf11b* encoding osteoprotegrin (OPG), a factor that binds to RANKL to block its actions by acting as a decoy receptor and in effect inhibit vascular calcification [23], [24] (Figure 3B). Taken together, these results showed that the EPA diet modulated the expression levels of *Mmp2* and *Mmp9*, which are involved in tissue remodeling, and *Tnfsf11* and *Tnfrsf11b*, which are involved in vascular wall calcification.

**Figure 4:**
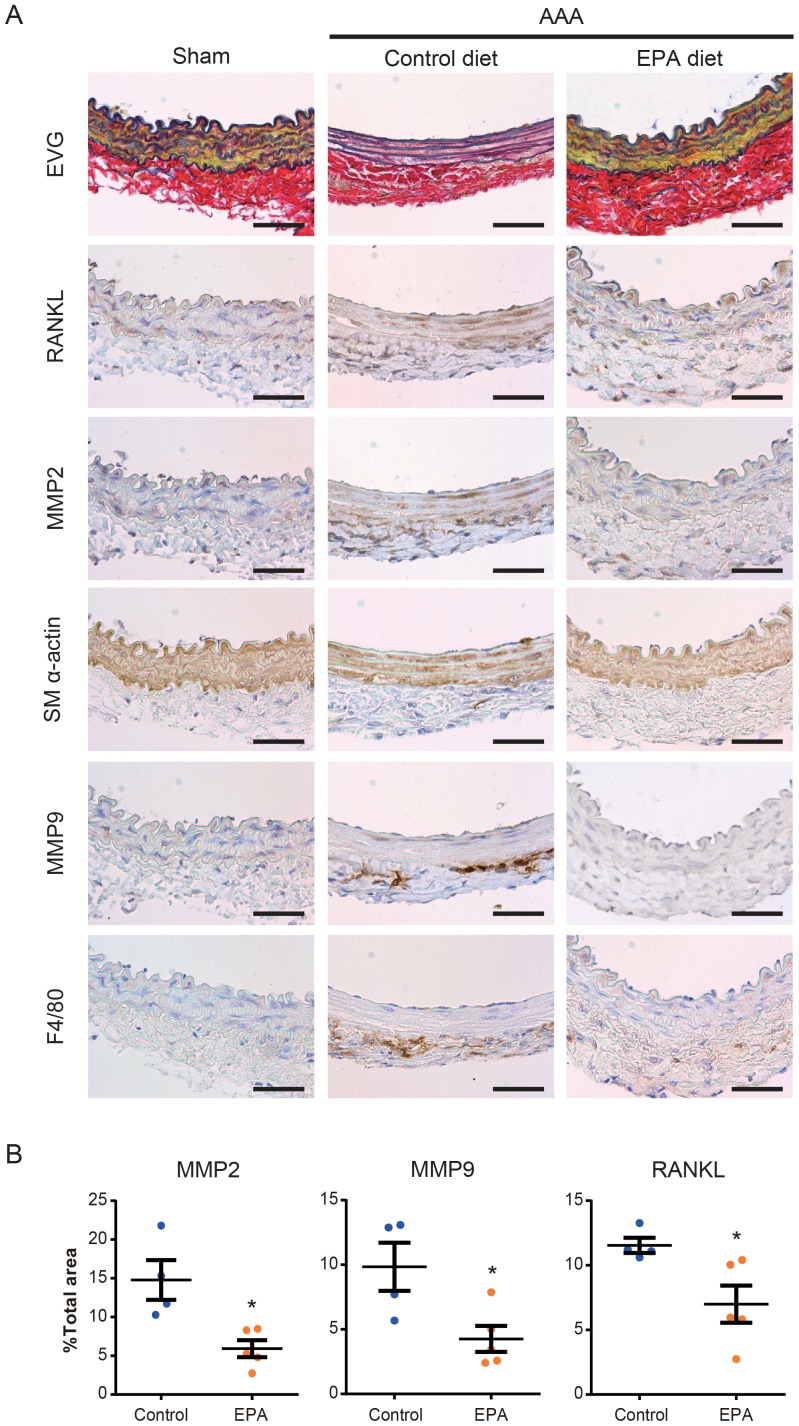
MMP2, MMP9, and RANKL expression in AAAs. **A**. Immunohistochemical staining for indicated proteins of serial sections of aortas one week after CaCl_2_ treatment. Elastic van Gieson staining is also shown. SM α-actin and F4/80 were stained to locate SMCs and macrophages, respectively. Shown are representative images of 4 or more samples in each group. Scale bars, 50 µm. **B**. Relative positive staining area of MMP2, MMP9, and RANKL in sections from control diet and EPA diet groups. *n* = 4–5. *P<0.05.

To further examine the cell-types expressing MMPs and RANKL, we performed immunohistochemical staining of these factors using serial sections of infra-renal aortas one week after CaCl_2_ treatment (Figure 4A). We found that MMP2 and RANKL shared similar localization patterns that mainly coincided with the staining of SM α-actin, a marker of SMCs. Some of the adventitial cells that stained positively for F4/80, a marker for macrophages, were also positive for MMP2 and RANKL. On the other hand, MMP9 co-localized predominantly with F4/80^+^ macrophages, consistent with previous reports. Quantification of the positive area of these factors showed a statistically significant reduction of MMP2, MMP9, and RANKL levels in the EPA diet group (Figure 4B), further corroborating the results of mRNA expression analyses.

### EPA suppresses *Mmp9* expression in macrophages

**Figure 5:**
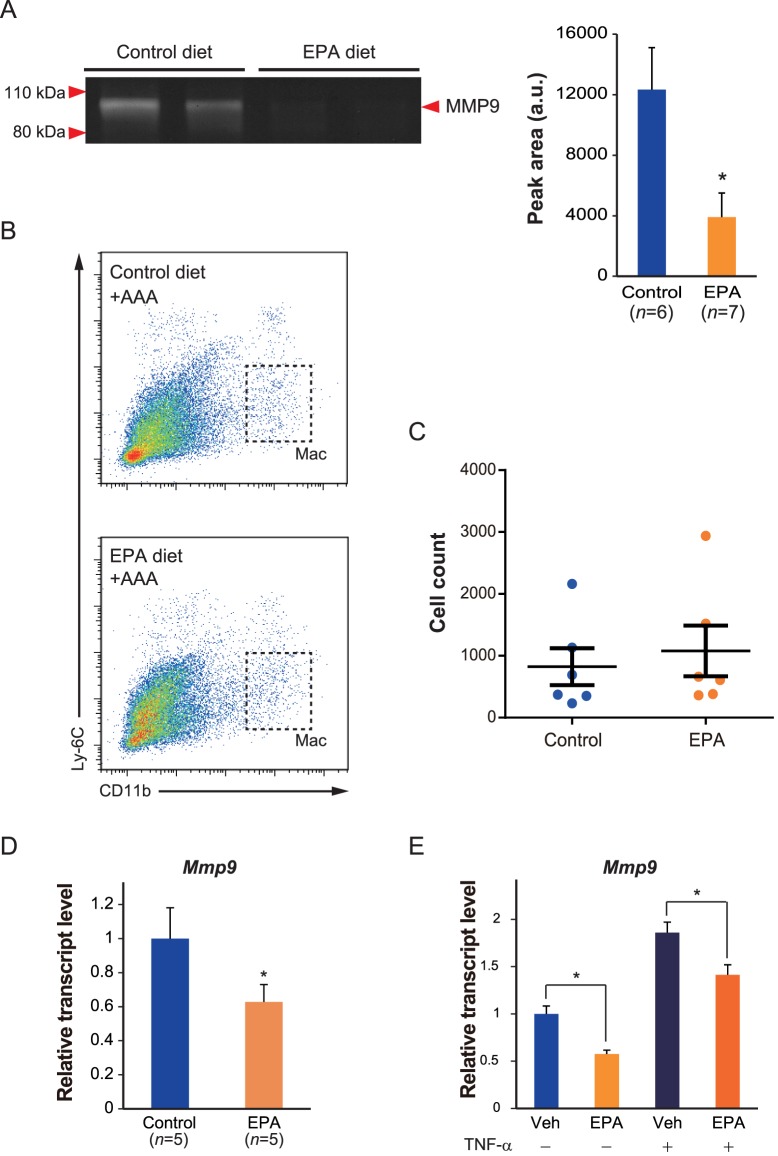
EPA reduces *Mmp9* expression in macrophages. **A**. Gelatin zymography of aortic tissues one week after CaCl_2_ treatment together with quantitative analysis, showing reduced MMP9 activity in samples from the EPA diet group. Equal amounts of protein (20 µg) were loaded per aortic sample. For quantitation, *n* = 6–7 in each group. **B**. Gating strategy for the flow cytometric analysis of AAA macrophages. Macrophages were identified as Ly-6C^low^CD11b^+^F4/80^+^Ly-6G^−^ cells (full gating strategy shown in Figure S2 in File S1). **C**. The number of aortic macrophages per aortic sample. No statistically significant difference in the number of aortic macrophages between control diet and EPA diet groups was detected. **D**. The mRNA levels of *Mmp9* in sorted aortic macrophages. Expression levels were first normalized to *18s* rRNA levels and then further normalized to the level of control diet group. *n* = 5 in each group. **E**. RAW264.7 macrophages were cultured with either vehicle (10% BSA) or EPA (50 µmol/L) for 48 hours. The cells were then stimulated with recombinant mouse TNF-α (20 ng/mL) for a further 6 hours and harvested for analysis by RT-PCR. Expression levels were first normalized to *18s* rRNA levels and then presented as relative expression compared to baseline vehicle sample. *n* = 3 per condition. *P<0.05 compared to control diet group in **A** and **D** or respective vehicle controls in **E**.

Previous reports have demonstrated that *Mmp9*-deficient mice are resistant to experimental AAA formation [6], [25]. Given that EPA seemed to impart a greater effect on *Mmp9* expression than on *Mmp2*, we decided to further analyze the possible involvement of MMP9 in EPA's effects on AAA formation. We performed gelatin zymography using aortic tissues one week after the CaCl_2_ treatment to examine the enzymatic activity of MMP9. Consistent with the results of mRNA levels, the aortas from the EPA diet group exhibited only about 30% of the MMP9 activity observed in the aortas from the control diet group (Figure 5A).

Macrophages have been reported to be the major producer of MMP9 in AAA tissues [6], [26], [27]. Our immunostaining results (Figure 4A) also showed that MMP9 was mainly expressed by macrophages. We therefore hypothesized that the decrease in MMP9 expression and activity might be due to a reduced number of macrophages recruited to the CaCl_2_-treated aortas of EPA-treated mice. To this end, we analyzed the number of macrophages in aortic tissues one week after the CaCl_2_ treatment by flow cytometry (Figure 5B, S2 in File S1). Contrary to our hypothesis, there was no significant difference in the number of Ly-6C^low^CD11b^+^F4/80^+^ macrophages [28] between the control diet and EPA diet groups (Figure 5C). However, when we subsequently sorted these macrophages and examined their *Mmp9* expression, there was significantly less *Mmp9* expressed by macrophages sorted from the aortas of EPA diet group (Figure 5D), suggesting that the EPA diet affected macrophage function, such as MMP production, within the aortic tissue.

To further address the possibility that EPA may directly inhibit macrophages from expressing *Mmp9*, we treated RAW264.7 macrophages with EPA *in vitro* and then further stimulated these cells with TNF-α to induce *Mmp9* expression. Consistent with the *in vivo* results, EPA attenuated the TNF-α-induced upregulation of *Mmp9* compared to vehicle control (Figure 5E). Taken together with the previous results, EPA appears to directly affect macrophages and reduce *Mmp9* expression.

### Osteogenic stimulation upregulates *Tnfsf11* as well as *Mmp2/9*

Our findings that EPA suppresses both AAA formation and calcification of aortic wall suggest that these two processes might be interlinked. To gain additional insight into this possibility, we analyzed the gene expression in SMCs under osteogenic conditions and found that stimulation with an osteogenic cocktail increased the levels of *Tnfsf11*, *Mmp2*, and *Mmp9* in cultured aortic SMCs (Figure S3 in File S1).

## Discussion

In this study, we demonstrated that the ω-3 PUFA, EPA, can attenuate AAA formation in a murine CaCl_2_-induced AAA model by suppressing tissue remodeling. Furthermore, EPA diet was also found to suppress vascular calcification in the model. In use clinically for over twenty years now, EPA alone or in combination with other ω-3 fatty acids has been shown to have pleiotropic benefits across a variety of diseases, such as the primary and secondary prevention of major coronary events [17], reduction of heart failure incidence [29], lowering blood pressure [30], improving outcomes of surgical and intensive care patients [31], and preserving renal function in patients with IgA nephropathy [32]. Further adding to these reports, our findings suggest that EPA may also be useful in slowing or preventing AAA formation.

Our results suggest that inhibition of *Mmp9* and *Mmp2* expression is one of the potential mechanisms by which EPA modulates tissue remodeling processes during AAA formation. In contrast to its effects on MMPs, EPA did not affect the expression of *Timp1* and *Timp2*, both of which are tissue inhibitors of a wide range of MMPs including MMP9 and MMP2 [33]. Given that the levels of both *Timp1* and *Timp2* in AAAs have also been shown to be associated with aneurysm formation [34], [35], it is likely that administration of EPA shifted the aortic microenvironment from a pro-proteolytic to an anti-proteolytic milieu by altering the balance between MMP9, MMP2, and TIMP levels.

While EPA did not reduce the accumulation of macrophages within CaCl_2_-treated aortic tissues under the experimental conditions of this study, it suppressed macrophage *Mmp9* expression (Figure 4, 5D). Previous studies have shown that genetic deletion of *Mmp9* inhibits CaCl_2_-induced AAA and that macrophages are the major source of MMP9 in AAAs [6], [26], [27]. Moreover, EPA inhibited TNF-α-induced expression of *Mmp9* in RAW264.7 macrophages (Figure 5E). Based on these results, it is likely that macrophages are one of the major cell-types that are directly affected by EPA in the aortic tissue. However, the expression of *Mmp2* was also modestly but significantly decreased by an EPA diet, demonstrated by the results of both gene expression (Figure 3A) and immunohistochemical (Figure 4) analyses. In the AAA milieu, MMP2 is considered to be primarily supplied by SMCs and fibroblasts and has also been shown in animal studies to be essential for the development of AAA [6], [36]. This was also demonstrated by our immunohistochemical staining results, where MMP2 predominantly localized to SM α-actin^+^ SMCs (Figure 4). Therefore, it appears that the effects of EPA on AAA formation may not simply be limited to macrophages. Indeed, our findings that EPA suppressed vascular calcification (Figure 2) and *Tnfsf11* (*Rankl*) expression (Figure 3B) in AAAs further suggest that EPA may also modulate the function of SMCs. Previous findings that EPA and DHA inhibited osteoblastic differentiation of a subpopulation of bovine medial cells [37] also support this notion. On the other hand, SMC-derived RANKL has been shown to recruit macrophages and promote their osteoclastic differentiation [38], suggesting that an interplay between macrophages and SMCs may promote vascular calcification and AAA formation. Furthermore, that an osteogenic cocktail induced the expression of factors necessary for vascular calcification (*Tnfsf11*) as well as MMPs (*Mmp2* and *Mmp9*) in SMCs (Figure S3 in File S1) suggest a link between vascular calcification and remodeling. Future studies are needed to further elucidate these complex interactions between vascular remodeling and calcification in AAAs and to assess the effects of EPA on these interactions.

Previous studies have shown that the renin-angiotensin system is important for AAA development, as demonstrated by the fact that angiotensin II infusion leads to AAA formation in *ApoE^−/−^* mice [39] as well as the fact that pharmacological inhibition of angiotensin II pathways have been shown to attenuate AAA development [40]. Thus, it will be interesting to investigate the effects of EPA on these models as well.

A number of pharmacological agents have been shown to suppress AAA formation in experimental animal models. Given their potential of limiting AAA progression, many of these agents, including statins, angiotensin-converting enzyme (ACE) inhibitors, antibiotics, beta blockers, and anti-inflammatory agents, are being investigated in clinical trials [40], [41]. However, results of most of the completed trials have been disappointing in that the studied medical treatments had either no or only marginal benefits in retarding aneurysm expansion [40], [42]–[45]. It is clear that future studies are needed to evaluate EPA's use in AAA prevention in humans, and the fact that EPA is already in clinical use widely, both as a nutritional supplement in the form of unpurified fish oil preparations and as a pharmacological agent in the form of ultra-purified EPA, should facilitate this.

## Materials and Methods

### Mice

Male 7 to 9 week-old BALB/cA mice were purchased from CLEA Japan (Tokyo) and kept in a temperature and humidity controlled room with a 12-hour light and 12-hour dark cycle. Mice were allowed unrestricted access to either a control diet (fish meal-free F1 chow, 362 kcal/100 g with 4.4% energy as fat; Funabashi Farm, Chiba) or an EPA-supplemented diet (fish meal-free F1 chow supplemented with 10% wt/wt EPA), and preparation of the diets has been described elsewhere [46], [47]. Ultrapure EPA was a generous gift from Mochida Pharmaceuticals Co., Ltd. (Tokyo). The CaCl_2_-induced AAA model was performed as previously described [6], [9]. Briefly, 4 days after the experimental diets were commenced, periaortic application of 0.5 mol/L CaCl_2_ (Sigma-Aldrich) for 15 minutes was performed in mice anaesthetized with intraperitoneal pentobarbital (IP) injection. After the procedure, the surgical wound was closed and the mice continued their experimental diets until sacrifice for analysis. At the 6-week time point, infrarenal aortas were photographed prior to harvesting and the external aortic diameter was determined by a blinded observer according to a previously described method [48].

### Ethics of Experimentation

All animal experiments were approved by the University of Tokyo Ethics Committee for Animal Experiments and strictly adhered to the guidelines for animal experiments of the University of Tokyo.

### Histological Analysis

Mice were perfused-fixed with 20% Tissue-Tek UFIX (Sakura Finetek Japan). The infra-renal aortas were then harvested, further fixed in 20% Tissue-Tek UFIX, dehydrated, embedded in paraffin, and 5 µm thick tissue slices were sectioned. Histological analysis was performed by Elastica van Gieson (EVG) and Masson's trichome staining according to standard procedures. Elastin breaks were quantified as previously described [49].

### Immunohistochemical Staining

Paraffin-embedded 5 µm thick serial aortic sections were treated with antigen retrieval solutions consisting of either 10 mmol/L Tris (pH 9.0) and 1 mmol/L EDTA (for MMP2, MMP9, and RANKL) or 0.1% trypsin (for F4/80). The sections were then blocked with 2% BSA for 10 minutes, treated with 3% H_2_O_2_ for 10 minutes, and then incubated with the following primary antibodies: rat anti-mouse F4/80 antibody (MCA497G, Serotec), mouse anti-actin, α-smooth muscle antibody (A2547, Sigma-Aldrich), goat anti-mouse MMP2 antibody (AF1488, R&D Systems), goat anti-mouse MMP9 antibody (AF909, R&D Systems), and goat anti-mouse RANKL antibody (AF462, R&D Systems). Sections were then incubated with biotinylated rabbit anti-goat secondary antibody (416021, Nichirei Bioscience) and developed with 3, 3′-diaminobenzadine (DAB). Positive areas for MMP2, MMP9, and RANKL of 3 sections per aortic sample were quantified with ImageJ software (National Institute of Health), and expressed as percentage positive area of total area examined.

### Micro-CT Imaging

Six weeks after induction of AAA formation by CaCl_2_, mice were anaesthetized with IP pentobarbital injections and IV contrast (ExiTron nano 6000, Miltenyi Biotec) was administered via the tail vein. The mice were then subjected to micro-CT imaging with the LaTheta LCT-200 CT scanner (Hitachi Aloka Medical, Ltd.). Quantification of aortic calcification was performed by taking 30 slices of the same section of infrarenal aorta in each animal and calculating the volume of calcification using the scanner's standard image analysis software.

### Quantitative Real-Time PCR Analysis

Total RNA was purified from aortic tissues or cultured cells using RNeasy kits (Qiagen), and using the RNeasy Plus Micro Kit (Qiagen) for cells sorted by flow cytometry, according to the manufacturer's instructions. Complementary DNA was synthesized using the SuperScript III First-Strand Synthesis System (Invitrogen). Quantitative real-time PCR analyses were conducted using the LightCycler system (Roche), with *18s* rRNA serving as the internal control. Primer sequences of the analyzed mouse genes were: *18s*, 5′-GCA ATT ATT CCC CAT GAA CG-3′ and 5′-GGG ACT TAA TCA ACG CAA GC-3′; *Mmp2*, 5′-TAA CCT GGA TGC CGT CGT-3′ and 5′-TTC AGG TAA TAA GCA CCC TTG AA-3′; *Mmp9*, 5′-ACG ACA TAG ACG GCA TCC A-3′ and 5′-GCT GTG GTT CAG TTG TGG TG-3′; *Timp1*, 5′-GCA AAG AGC TTT CTC AAA GAC C-3′ and 5′-AGG GAT AGA TAA ACA GGG AAA CAC T-3′; *Timp2*, 5′-CGT TTT GCA ATG CAG ACG TA-3′ and 5′-GGA ATC CAC CTC CTT CTC G-3′; *Tnfrsf11b*, 5′-GTT TCC CGA GGA CCA CAA T-3′ and 5′-CCA TTC AAT GAT GTC CAG GAG-3′; *Tnfsf11*, 5′-TGA AGA CAC ACT ACC TGA CTC CTG-3′ and 5′-CCA CAA TGT GTT GCA GTT CC-3′. Sequences of the rat primers were: *18s*, 5′-CGA AAG CAT TTG CCA AGA AT-3′ and 5′-AGT CGG CAT CGT TTA TGG TC-3′; *Mmp2*, 5′-CAC CAC CGA GGA TTA TGA CC-3′ and 5′-CAC CCA CAG TGG ACA TAG CA-3′; *Mmp9*, 5′-CCT CTG CAT GAA GAC GAC ATA A-3′ and 5′-GGT CAG GTT TAG AGC CAC GA-3′; *Tnfsf11*, 5′-CAT CGG GTT CCC ATA AAG-3′ and 5′-GAA GCA AAT GTT GGC GTA-3′. The primer sequence for rat *Tnfsf11* was previously described [50]. All other primer sequences were designed with the Roche Universal ProbeLibrary Assay Design Center.

### Zymography

Infra-renal aortas from control or EPA diet-fed mice were harvested and placed immediately into liquid nitrogen. The frozen samples were homogenized in 2× lysis buffer containing 50 mmol/L Tris/HCl (pH 7.5), 150 mmol/L NaCl, 1.0% IGEPAL CA-630, 2 mmol/L EDTA that was combined in a 1∶1 ratio with 25× cOmplete EDTA-free protease inhibitor cocktail (Roche Diagnostics). The homogenate was briefly centrifuged at 4°C and the supernatant containing protein was used for analysis. Protein concentration of each aortic extract was determined with the DC Protein Assay (Bio-Rad). Zymography was performed as previously described [51]. Briefly, 20 µg of total protein was equally loaded onto each well of a Novex 10% Zymogram (gelatin) gel (Invitrogen) and separated under non-reducing conditions. The gels were then renatured and developed, followed by staining with SimplyBlue SafeStain (Life Technologies) and destaining in fresh deionized water.

### Flow Cytometric Analysis and Cell Sorting

Three fresh, isolated infra-renal aortas from the same experimental group were pooled into one sample for flow cytometric analysis. Pooled samples were first cut into fine pieces on ice, and then dissociated into individual cells by incubating samples in Hank's balanced salt solution (with Ca^2+^ and Mg^2+^) containing 400 U/mL collagenase type II (Worthington Labs), 0.75 U/mL elastase (Worthington Labs), and 60 U/mL DNase I (Sigma-Aldrich) at 37°C for one hour with shaking. After incubation, the cells were passed through a Falcon 100 µm Cell Strainer (BD Japan), centrifuged at 1500 rpm, 4°C for 5 minutes, and cell pellets were resuspended in ice cold FACS buffer (PBS containing 5% FBS) after discarding the supernatant. Cell pellets were washed at least twice with FACS buffer, followed by flow cytometric analysis. The antibodies used for analysis were anti-CD11b (clone M1/70) from eBioscience; anti-F4/80 (BM8), anti-Ly-6C (HK1.4), and anti-Ly-6G (1A8) from BioLegend. Corresponding isotype controls for each antibody were also used. Dead and Ly-6G-negative aortic cells were gated out, and the remaining cells were subjected to further analyses using FACSAria II (BD).

### Cell Culture

Murine RAW264.7 macrophages were obtained from American Type Culture Collection and cultured in DMEM (Gibco) with complete supplementation consisting of 10% FBS (Hyclone), 0.68 mmol/L L-glutamine, 100 units/mL penicillin (Life Technologies), and 100 µg/mL streptomycin (Life Technologies). Stock solutions of 150 mmol/L EPA (Cayman Chemical) were prepared and stored according to the manufacturer's instructions until use. Culture medium containing EPA was prepared according to previously described methods for fatty acid preparation, with some minor modifications [52]. Briefly, aliquots of the stock solution of EPA were complexed with fatty-acid-free, low-endotoxin BSA (10% wt/vol solution in H_2_O, Sigma-Aldrich) to give a 7.5 mmol/L working solution, which was incubated at 37°C for 30 minutes. After incubation, the working solution was added to warmed DMEM with complete supplementation to give a final concentration of 50 µmol/L. The vehicle solution was prepared similarly using a mixture of ethanol/water instead of EPA, and this was used as the control. Twenty ng/mL of recombinant mouse TNF-α protein (R&D Systems) was added after 48 hours of incubating cells with vehicle- or EPA-containing medium. The methods for culturing rat aortic SMCs were described previously [53]. Smooth muscle cells were cultured in DMEM/F12 (Gibco) supplemented with 10% FBS and the aforementioned antibiotics. For osteogenic stimulation, confluent cells were first cultured in a defined serum-free medium [53] for 3 days, after which a modified osteogenic cocktail [54] was added to give a final concentration of 1 µmol/L ascorbic acid (Sigma-Aldrich), 10 mmol/L β-glycerophosphate (Sigma-Aldrich), and 10 nmol/L dexamethasone (Sigma-Aldrich). Medium was changed every 3 to 4 days with concurrent addition of fresh osteogenic cocktail.

### Statistical Analysis

All data are shown as means ± SEM. Differences between two groups were analyzed using Student's *t*-test, while differences between three or more groups were analyzed using one-way ANOVA followed by Tukey's post-hoc test. *P* values of less than 0.05 were considered to be statistically significant. All statistical analyses were performed using GraphPad Prism 5 software.

## Supporting Information

File S1 **Supporting Information.** **Figure S1.** Fibrosis in CaCl2-induced AAA. Histological analysis of the degree of aneurysmal fibrosis with Masson's Trichrome staining at 6 weeks after periaortic application of CaCl2, showing increased medial fibrosis in the AAA of control diet group compared to the EPA diet group. In general, there was no difference in adventitial fibrosis between the two groups. Connective tissue (e.g. collagen) stain blue while muscle cells stain red. Scale bars, 200 µm (upper panels) and 50 µm (lower panels). Images are representative of at least three independent experiments. **Figure S2.** Gating strategy for the flow cytometric analysis of aortic macrophages. Living cells isolated from aortic tissues were first gated on Ly-6G (granulocyte marker), and Ly-6G^−^ cells were further analyzed for expression of Ly-6C and CD11b (**A**); Ly-6C^low^CD11b^+^ cells were shown to be positive for F4/80, a macrophage marker (**B**), and Ly-6C^low^CD11b^+^F4/80^+^ cells were taken to be aortic macrophages and used in all subsequent analyses. **C**. Giemsa staining of sorted Ly-6C^low^CD11b^+^F4/80^+^ cells from the aorta shows cells with the characteristic macrophage appearance. Scale bar, 10 µm. **Figure S3**. Stimulation of vascular SMCs with osteogenic cocktail induces the expression of osteogenic and tissue remodeling factors. Rat primary vascular SMCs were stimulated with an osteogenic cocktail (OC) for 7 days. Expression of the osteogenic factor *Tnfsf11* (RANKL) and tissue remodeling factors *Mmp2* and *Mmp9* were analyzed using real-time PCR. *n* = 3 per condition. *P<0.05.
(PDF)
