# Supplementary material for: The ω-3 Polyunsaturated Fatty Acid, Eicosapentaenoic Acid, Attenuates Abdominal Aortic Aneurysm Development via Suppression of Tissue Remodeling
Source: PLoS One. 2014 May 5;9(5):e96286. doi: 10.1371/journal.pone.0096286 (PMC4010435; doi:10.1371/journal.pone.0096286)
Supplement: File S1 — Supporting Information. Figure S1. Fibrosis in CaCl2-induced AAA. Histological analysis of the degree of aneurysmal fibrosis with Masson's Trichrome staining at 6 weeks after periaortic application of CaCl2, showing increased medial fibrosis in the AAA of control diet group compared to the EPA diet group. In general, there was no difference in adventitial fibrosis between the two groups. Connective tissue (e.g. collagen) stain blue while muscle cells stain red. Scale bars, 200 µm (upper panels) and 50 µm (lower panels). Images are representative of at least three independent experiments. Figure S2. Gating strategy for the flow cytometric analysis of aortic macrophages. Living cells isolated from aortic tissues were first gated on Ly-6G (granulocyte marker), and Ly-6G− cells were further analyzed for expression of Ly-6C and CD11b (A); Ly-6ClowCD11b+ cells were shown to be positive for F4/80, a macrophage marker (B), and Ly-6ClowCD11b+F4/80+ cells were taken to be aortic macrophages and used in all subsequent analyses. C. Giemsa staining of sorted Ly-6ClowCD11b+F4/80+ cells from the aorta shows cells with the characteristic macrophage appearance. Scale bar, 10 µm. Figure S3. Stimulation of vascular SMCs with osteogenic cocktail induces the expression of osteogenic and tissue remodeling factors. Rat primary vascular SMCs were stimulated with an osteogenic cocktail (OC) for 7 days. Expression of the osteogenic factor Tnfsf11 (RANKL) and tissue remodeling factors Mmp2 and Mmp9 were analyzed using real-time PCR. n = 3 per condition. *P<0.05. (PDF) [file pone.0096286.s001.pdf]

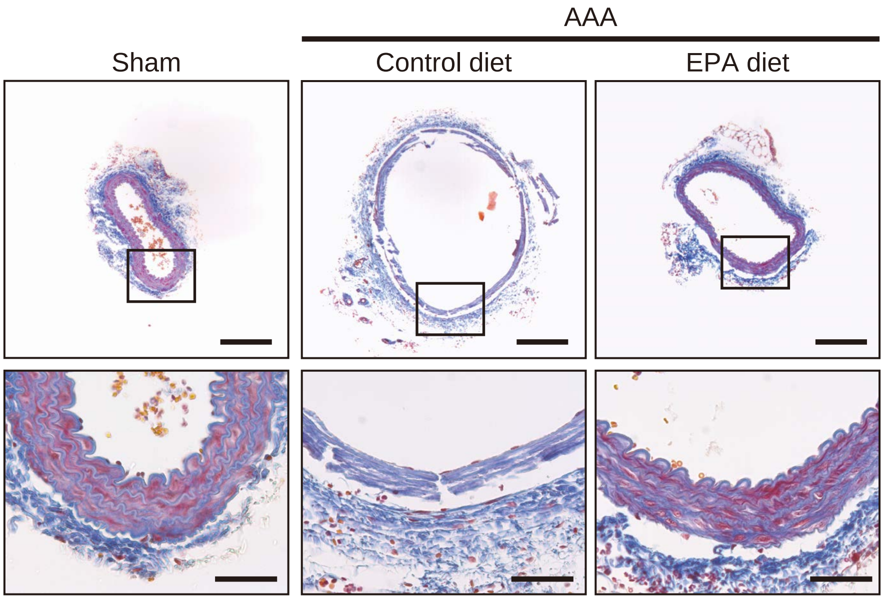

Figure S1. Fibrosis in $CaCl_2$-induced AAA.

Histological analysis of the degree of aneurysmal fibrosis with Masson's Trichrome staining at 6 weeks after periaortic application of $CaCl_2$, showing increased medial fibrosis in the AAA of control diet group compared to the EPA diet group. In general, there was no difference in adventitial fibrosis between the two groups. Connective tissue (e.g. collagen) stain blue while muscle cells stain red. Scale bars, 200 μm (upper panels) and 50 μm (lower panels). Images are representative of at least three independent experiments.

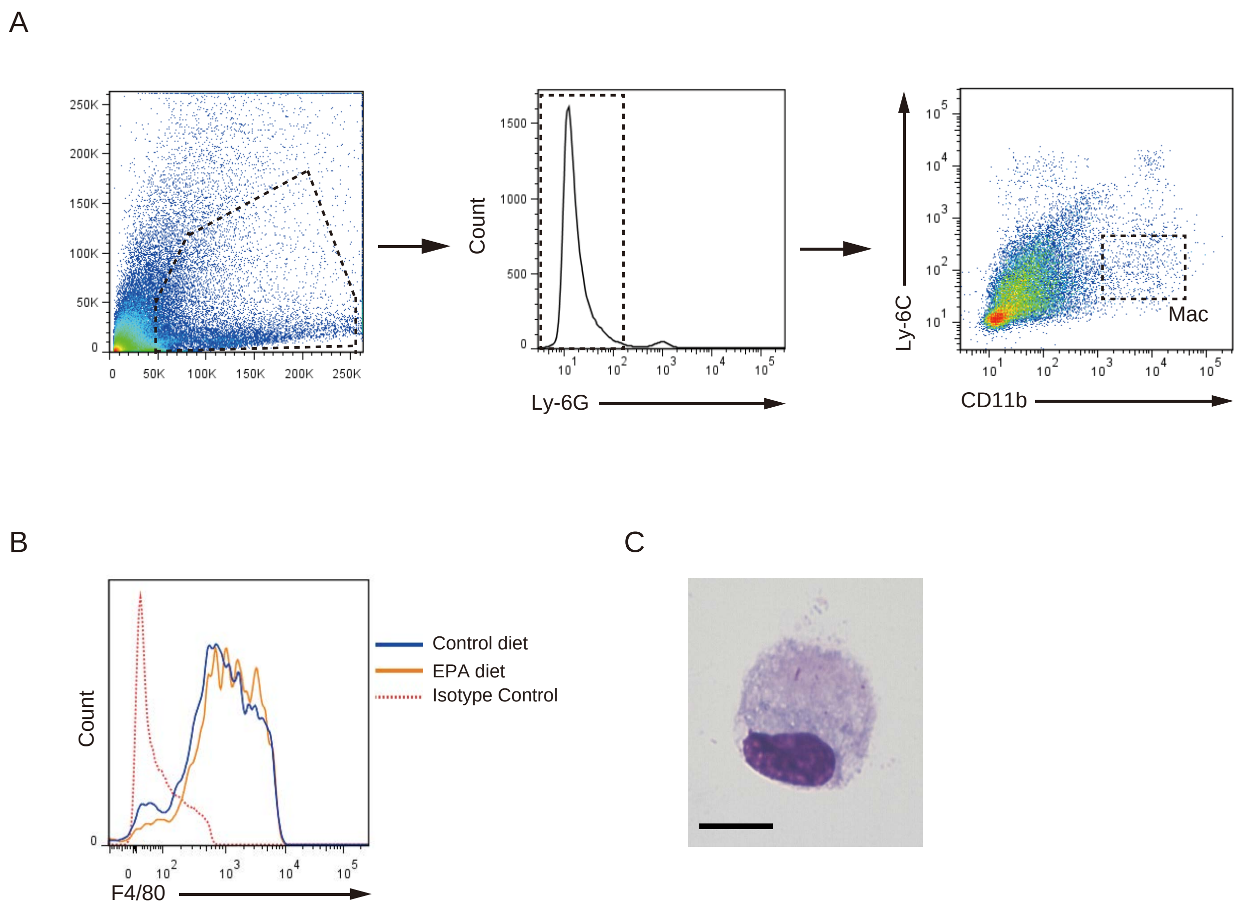

Figure S2. Gating strategy for the flow cytometric analysis of aortic macrophages.

Living cells isolated from aortic tissues were first gated on Ly-6G (granulocyte marker), and Ly-6G^{-} cells were further analyzed for expression of Ly-6C and CD11b **(A)**; Ly-6C^{low}CD11b^{+} cells were shown to be positive for F4/80, a macrophage marker (**B)**, and Ly-6C^{low}CD11b^{+}F4/80^{+} cells were taken to be aortic macrophages and used in all subsequent analyses. **C.** Giemsa staining of sorted Ly-6C^{low}CD11b^{+}F4/80^{+} cells from the aorta shows cells with the characteristic macrophage appearance. Scale bar, 10 µm.

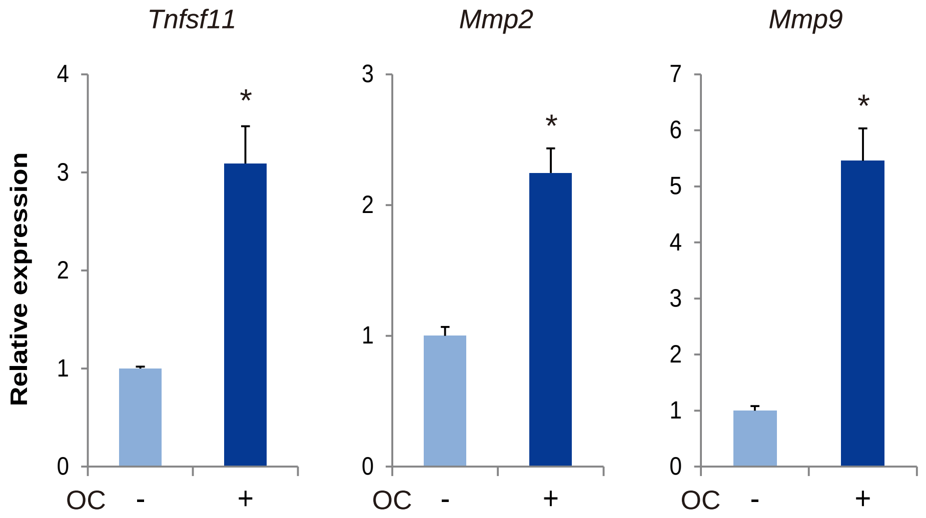

Figure S3. Stimulation of vascular SMCs with osteogenic cocktail induces the expression of osteogenic and tissue remodeling factors.

Rat primary vascular SMCs were stimulated with an osteogenic cocktail (OC) for 7 days. Expression of the osteogenic factor *Tnfsf11* (RANKL) and tissue remodeling factors *Mmp2* and *Mmp9* were analyzed using real-time PCR. $n = 3$ per condition. *$P < 0.05$.
